# Effect of Graphene and Graphene Oxide on Airway Barrier and Differential Phosphorylation of Proteins in Tight and Adherens Junction Pathways

**DOI:** 10.3390/nano11051283

**Published:** 2021-05-13

**Authors:** Sofie Van Den Broucke, Jeroen A. J. Vanoirbeek, Rita Derua, Peter H. M. Hoet, Manosij Ghosh

**Affiliations:** 1 Centre for Environment and Health, Department of Public Health and Primary Care, KU Leuven, 3000 Leuven, Belgium; vandenbroucke.sofie@gmail.com; 2 Laboratory of Respiratory Diseases and Thoracic Surgery (BREATHE), KU Leuven, 3000 Leuven, Belgium; jeroen.vanoirbeek@kuleuven.be; 3 Laboratory of Protein Phosphorylation and Proteomics, KU Leuven, 3000 Leuven, Belgium; rita.derua@kuleuven.be

**Keywords:** tight junction, adherens junction, phosphoproteomics, graphene nanoparticles, barrier integrity

## Abstract

Via inhalation we are continuously exposed to environmental and occupational irritants which can induce adverse health effects, such as irritant-induced asthma (IIA). The airway epithelium forms the first barrier encountered by these agents. We investigated the effect of environmental and occupational irritants on the airway epithelial barrier in vitro. The airway epithelial barrier was mimicked using a coculture model, consisting of bronchial epithelial cells (16HBE) and monocytes (THP-1) seeded on the apical side of a permeable support, and human lung microvascular endothelial cells (HLMVEC) grown on the basal side. Upon exposure to graphene (G) and graphene oxide (GO) in a suspension with fetal calf serum (FCS), ammonium persulfate (AP), sodium persulfate (SP) and hypochlorite (ClO^−^), the transepithelial electrical resistance (TEER) and flux of fluorescent labelled dextran (FD4-flux), was determined. Exposure to graphene nanoparticles (GNPs) induced an immediate negative effect on the epithelial barrier, whereas ClO^−^ only had a negative impact after 24 h of exposure. AP and SP did not affect the barrier properties. The tight junctions (TJ) network showed less connected zonula occludens 1 (ZO-1) and occludin staining in GNP-exposed cocultures. Functional analysis of the phosphoproteomic data indicated that proteins in the adherens junction (AJ) and TJ pathways showed an altered phosphorylation due to GNP exposure. To conclude, the negative effect of GNPs on the epithelial barrier can be explained by the slightly altered the TJ organization which could be caused by alterations in the phosphorylation level of proteins in the AJ and TJ pathway.

## 1. Introduction

The airway epithelium is located at the interface between the internal and external environment and therefore plays an important role in host defense [1]. Via intercellular junctional complexes, the airway epithelial cells are tightly connected and create a first barrier to the outside world. Intercellular junctions are dynamic structures that confer a loose structure when ions, solutes or immune cells have to pass the epithelial layer, yet also establish an impenetrable fence to harmful agents [2,3]. Intercellular junctional complexes consist of apically localized tight junctions (TJ) which regulate paracellular transport of ions and small molecules and establish an apical–lateral polarity, and the underlying adherens junctions (AJ) which are important for initiation and maintenance of cell–cell adhesion. TJs are made up of adhesive transmembrane proteins (e.g., claudin, occludin and junction adhesion molecule (JAM) families), actin-binding cytoplasmic proteins (e.g., zonula occludens (ZO) proteins 1, 2 and 3) and associated scaffolding proteins. The principal proteins in AJs are the cadherin and nectin transmembrane protein family and intracellular β-catenin, α-catenin and p120 catenin [4,5].

Various types of environmental and occupational agents, such as allergens, pollution particles and respiratory tract viruses can impair the epithelial barrier integrity by disturbing the intercellular junctional complexes, and therefore lead to detrimental health consequences [4,5]. Dysregulation of the TJ and AJ has been implicated in a variety of pulmonary conditions, including asthma, rhinosinusitis and COPD [6,7,8]. In the current study, we aimed to investigate the effect of different types of environmental and occupational irritants on the airway epithelium. Exposure to respiratory irritants, either at a high dose or repeatedly lower doses, can result in the development of an asthmatic syndrome termed irritant-induced asthma (IIA) [9,10]. Because the airway epithelium is the first line of cells that come into contact with respiratory irritants, its response upon irritant exposure might provide insight in the first steps towards development to IIA.

Since their discovery, graphene nanoparticles (GNPs) have gained considerable attention and research and applications have emerged rapidly [11,12]. Graphene consists of a single-atom thick, two-dimensional sheet of carbon atoms, resulting in a unique platelet-like shape. The GNP sheets are able to penetrate deep into the lungs, where they pose a possible risk for respiratory health [13,14]. Various studies investigated the toxicity of GNPs in vitro, however their effect on the epithelial barrier has not been studied before [13,15,16,17,18]. In this study, the effect induced by GNPs were compared with that of known irritants-hypochlorite and persulfate salts. Hypochlorite (ClO^−^) is a chlorine derivative, used as household bleach and swimming pool disinfectant, which can exert a variety of respiratory symptoms, including IIA [19] through direct contact with the respiratory epithelium mediated by oxidative tissue injury [20,21]. Persulfate salts are an important component of hair bleaching powder [22]; and have been identified as the main causal agents in occupational asthma in hairdressers, with limited information on the mechanism of the hypersensitive airway disorder [23,24,25,26].

## 2. Materials and Methods

### 2.1. Cell Culture

The human bronchial epithelial cells, 16HBE14o- (16HBE) and THP-1 cells (a human acute monocytic cell line), were cultured in DMEM/F12 and RPMI medium, respectively [27,28]. Human lung microvascular endothelial cells (HLMVEC) were cultured in human lung microvascular endothelial cell growth medium [27]. The medium was renewed every 2–3 days, and when confluent, the 16HBE and HLMVEC cells were released enzymatically (0.05% trypsin-EDTA) and transferred to a new culture flask [27,28].

### 2.2. Exposure Conditions

**Figure 1:**
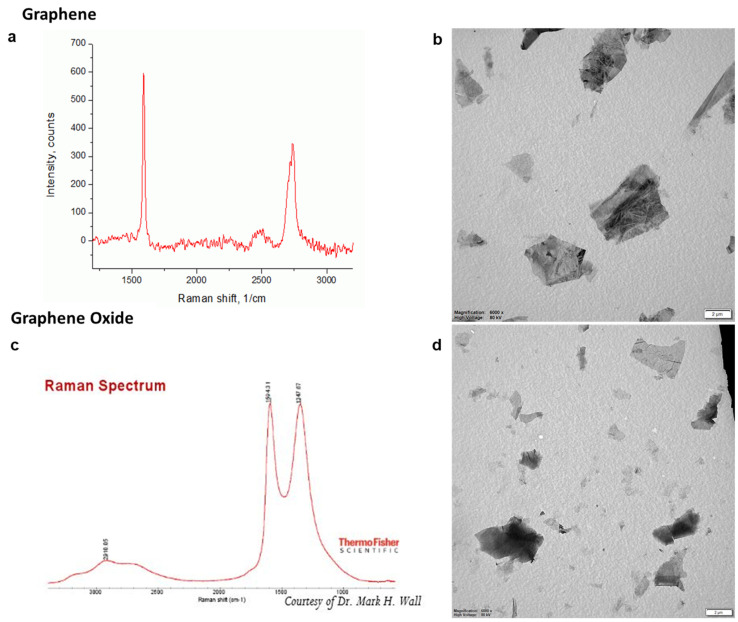
Characterization of G and GO NPs—Raman spectrum provided from the manufacturer of (**a**) G and (**c**) GO. Representative transmission electron spectroscopy (TEM) images of (**b**) G and (**d**) GO.

Graphene nanoparticles (GNPs) were purchased from Graphene-supermarket.com. Graphene (G) has a lateral dimension of 2 to 8 µm and consists of 3 to 8 graphene monolayers based on the manufacturer’s description. Graphene oxide (GO) has a flake size of 0.5 to 5 µm and contains 80% of graphene oxide monolayers. Figure 1 shows the Raman spectra of G and GO provided from the manufacturer. For G, the high intense peak at 1583 cm^−1^ (G band) and a low intense peak between 2500 and 3000 cm^−1^ (2D band) indicate that the provided G consists of multiple layers of graphene (Figure 1a). The Raman spectrum of GO shows the graphene characterizing peak at 1586 cm^−1^ (G band) and a peak between 1270 and 1450 cm^−1^ (D band) that indicates the different structure in the graphene layer due to the oxygen binding (Figure 1c). Transmission electron microscopy (TEM) images of the G and GO particles in suspension with 2% FCS are shown in Figure 1. The particle dimensions stated by the manufacturer correspond to the dimensions observed on the acquired TEM images. Based on the ‘darkness’ of the particles, it can be visualized that the G sheets mostly consisted of multiple layers, and few monolayers were present (Figure 1b). For GO, aggregated monolayers as well as monolayers were observed (Figure 1d). The levels of endotoxin in the sample suspensions were evaluated using the Endosafe^®^-PTS Limuluss Amoebocyte Lysate (LAL) assay according to the manufacturer’s instructions. The sensitivity of the assay we used was 0.05 EU/mL.

The GNPs were weighed and dispersed in sterile water (Baxter) containing 2 vol% FCS (= dispersion medium) at a concentration of 2.56 mg/mL. These stock suspensions were dispersed using a Microson ultrasonic cell disruptor (Microsonix, Newton, MA, USA), equipped with a disruptor horn, in a glass beaker on ice for 16 min at 730 watts [28]. Intermediate concentrations were made in the dispersion medium and afterwards diluted tenfold in the culture medium to obtain final concentrations.

The levels of endotoxin in the sample suspensions were evaluated using the Endosafe^®^-PTS Limuluss Amoebocyte Lysate (LAL) assay according to the manufacturer’s instructions. The sensitivity of the assay we used was 0.05 EU/mL [28].

To prepare the final ammonium persulfate (AP) and sodium persulfate (SP) solutions for the experiments, AP and SP salts were weighed and dissolved in culture medium containing 0.2% FCS. Sodium hypochlorite with 13% active chlorine (act. Cl) was dissolved and diluted in dispersion medium, to obtain final concentrations that are expressed as parts per million (ppm) of act. Cl in the final hypochlorite (ClO^−^) solution.

### 2.3. Cytotoxicity Measurements

In order to determine sub-toxic concentrations for exposure in the coculture, two different cytotoxicity assays were performed on 16HBE monocultures. The cells were seeded in a 96-well plate at a density of 200,000 cells/cm^2^. The next day, the cells were washed with serum-free culture medium and subsequently exposed to increasing concentrations of G, GO, AP, SP and ClO^−^. After the incubation period of 24 h, the lactate dehydrogenase (LDH) and the 2-(4-Iodophenyl)-3-(4-nitrophenyl)-5-(2,4-disulfophenyl)-2H-tetrazolium (WST-1) assay, cytotoxicity was estimated according to methods previously described [28].

### 2.4. Coculture

#### 2.4.1. Setup

A coculture model of the lung–blood barrier, using human bronchial epithelial cells (16HBE14o-), monocytes (THP-1) and human lung microvascular endothelial cells (HLMVEC) was established according to method described previously [27,28]. Briefly, the HLMVECs were seeded the on the lower side of a non-coated polyester Transwell^®^ insert membrane (0.4 µm pore density and 0.33 cm^2^ surface area, Sigma-Aldrich, Overijse, Belgium) followed by seeding of 16HBE cells on the upper side. A total of 4 days after seeding, the THP-1 cells were added to the apical compartment in a 1/10 ratio with the 16HBE cells. The medium in the apical compartment consisted of a mix of 16HBE (9/10) and THP-1 (1/10) medium with 0.2% FCS [27,28].

#### 2.4.2. Exposure

On day 5 in culture, the cocultures were exposed on the apical compartment of the transwell insert. Concentrations of 64 or 256 µg/mL for G, 16 or 64 µg/mL for GO, 25 or 100 µg/mL for AP or 12.5 or 6.25 µg/mL for SP and 7.5 or 3.75 ppm act. Cl in ClO^−^ in a medium containing a final concentration of 0.2% FCS were used. Control cocultures were treated with the vehicle culture medium containing 0.2% FCS.

#### 2.4.3. Measurement of the Transepithelial Electrical Resistance (TEER)

The transepithelial electrical resistance (TEER) was measured using the Evom Voltohmmeter supplemented with the End-Ohm chamber or STX2 chopstick (World Precision Instruments Inc., Sarasota, FL, USA), at 30 min, 1, 2, 4, 6 and 24 h after the start of exposure, as previously described [27].

#### 2.4.4. Paracellular Flux of Fluorescent Labelled Dextran

Paracellular flux of uncharged molecules was investigated by measuring passage of apically added markers across the cell layer as previously described [28]. Fluorescein isothiocyanate (FITC)-conjugated 4 kDa-dextran (46,944 Sigma-Aldrich, Overijse, Belgium) was added to the apical surface at a final concentration of 1 mg/mL. After 4 h of incubation at 37 °C, triplicate basolateral samples were taken for assaying (excitation and emission wavelengths of 485 and 530 nm) [28] on a black 96-well plate. The flux or change in concentration was calculated by dividing the concentration and volume of the basolateral compartment by the concentration and volume of the apical compartment and expressed as a percentage.

#### 2.4.5. Staining of the Intercellular Junctional Proteins

Integrity of the tight junction was assessed using immunofluorescent staining of proteins zonula occludens 1 (ZO-1) and occludin. After the exposed cocultures were washed, fixed with ice-cold methanol for 10 min at −20 °C and permeabilized with 0.3% Triton-X100 for 10 min, the coculture was blocked with 1% BSA solution for 1 h and incubated successively with Alexa Fluor^®^ 488-conjugated mouse anti-human ZO1 antibody (1/100, ThermoFisher, Gent, Belgium) and Alexa Fluor^®^ 594-conjugated mouse anti-human Occludin antibody (1/100, ThermoFisher, Gent, Belgium) for 2 h in 0.5% BSA solution. The nuclei were counterstained with DAPI (1/500) for 10 min. Finally, the membranes were excised from the insert and mounted using ProLong^®^ Gold Antifade Mountant (ThermoFisher, Gent, Belgium). Images were taken with an Olympus BX61 microscope using the XC30 camera at ×40 magnification.

### 2.5. Phosphoproteomics

16HBE cells were exposed to 64 mg/mL of G or GO for 24 h, and after this the cells were washed and lysed using the Mem-PER™ Plus membrane protein extraction kit (ThermoFisher, Gent, Belgium) supplemented with 1 mM Phenylmethylsulfonyl fluoride (PMSF) and phosSTOP™ (Sigma-Aldrich, Overijse, Belgium) to extract the membrane-associated proteins, according to the manufacturers’ instructions. Membrane protein concentrations were determined using the Pierce bicinchoninic acid (BCA) protein assay (ThermoFisher, Gent, Belgium).

A total of 180 µg of membrane fraction lysates were subjected to reduction (5 mM DTT) and alkylation (25 mM iodoacetamide) followed by precipitation [29]. The proteins were digested overnight using 4 µg trypsin at 37 °C in 200 mM ammonium bicarbonate, 5% acetonitrile, 0.01% ProteaseMax (Promega, Belgium). The resulting peptides were desalted with C18 Spin Columns (Harvard Apparatus, Holliston, MA, USA) and subjected to phosphopeptide enrichment on IMAC beads (PHOS-Select iron affinity gel, Sigma-Aldrich, Overijse, Belgium). The resulting peptides were desalted with C18 ZipTip pipette tips (Millipore) and subjected to high-resolution LC-MS/MS using an Ultimate 3000 nano UPLC system interfaced with a Q Exactive hybrid quadrupole-orbitrap mass spectrometer via an EASY-spray (C-18, 50 cm) column (Thermo Fisher Scientific) [30].

Progenesis software (Nonlinear Dynamics) was used for relative quantification of peptides. Peptides were identified by MASCOT (Matrix Science) using SwProt_trEMBL_homo sapiens (71,785 sequences) as a database via Proteome Discoverer 2.2 software, incorporating Percolator for peptide validation and ptmRS for PTM localization. The following search parameters were used—phosphorylation (STY) in combination with oxidation (M) as variable modifications and carbamidomethylation (C) was used as a fixed modification. Three missed cleavages were allowed for trypsin digestion. Peptide tolerance was set at 10 ppm and MS/MS tolerance at 20 mmu. Only peptides with a high (PEP < 0.01) and middle (PEP < 0.05) identification confidence were considered.

The peptide and protein abundancies of the NP-exposed cells were divided by those of the control cells, to quantify the change in phosphorylation. If the ratio of phosphopeptide abundance of the NP-exposed cells compared to that of the control cells was larger or equal than 1.5, or smaller or equal than 0.5, these proteins were considered as relevantly hyper- or hypophosphorylated. These unique proteins were selected as per the type of NP and were further investigated. Functional analysis of the selected proteins was performed using the DAVID web tool for their involvement in cell pathways using the Kyoto Encyclopedia of Genes and Genomes (KEGG) database (https://www.genome.jp/kegg/; accessed on 26 June 2018) [31,32,33]. As a background, the DAVID-supported ‘Homo sapiens’ gene set was used.

### 2.6. Statistical Analysis

The results are represented as mean ± SD or mean with individual values. Normality distribution was determined using the d’Agostino and Pearson omnibus normality test. When normally distributed, the data were analyzed with a one-way ANOVA followed by Dunnett’s multiple-comparison test. When the data were not normally distributed, the Kruksal–Wallis test followed by a Dunn’s multiple comparison test was used. Time series of the TEER measurements were analyzed with a two-way ANOVA (Graphpad Prism 5.00, Graphpad Software Inc., San Diego, CA, USA). A level of *p* < 0.05 was considered significant [28].

## 3. Results

### 3.1. Effect on Transepithelial Electrical Resistance (TEER)

**Figure 2:**
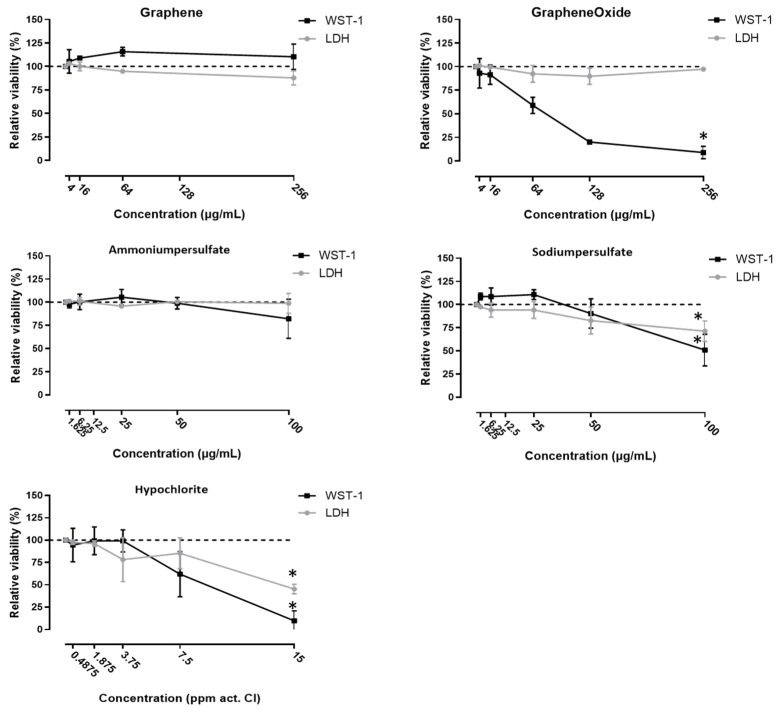
WST-1 and LDH assays of 16HBE cells exposed to G, GO, AP, SP and ClO^−^—the 16HBE cells were exposed to increasing concentrations of the agents for 24 h. Afterwards, the cell viability was evaluated using the WST-1 and LDH assay. Mean ± SD, *n* = 2–3, each experiment was performed in triplicate. * *p* < 0.05 (*t*-test).

Changes in ion permeability of the cell layers due to irritant exposure were examined using measurement of the transepithelial electrical resistance (TEER). Cocultures were apically exposed to 64 or 256 µg/mL G, 16 or 64 µg/mL GO, 25 or 100 µg/mL AP, 12.5 or 6.25 µg/mL SP, 7.5 or 3.75 ppm act. Cl in ClO^−^, or fresh medium (Ctrl). These concentrations were sub-toxic for 16HBE cells, as determined by LDH and WST-1 assay (Figure 2).

**Figure 3:**
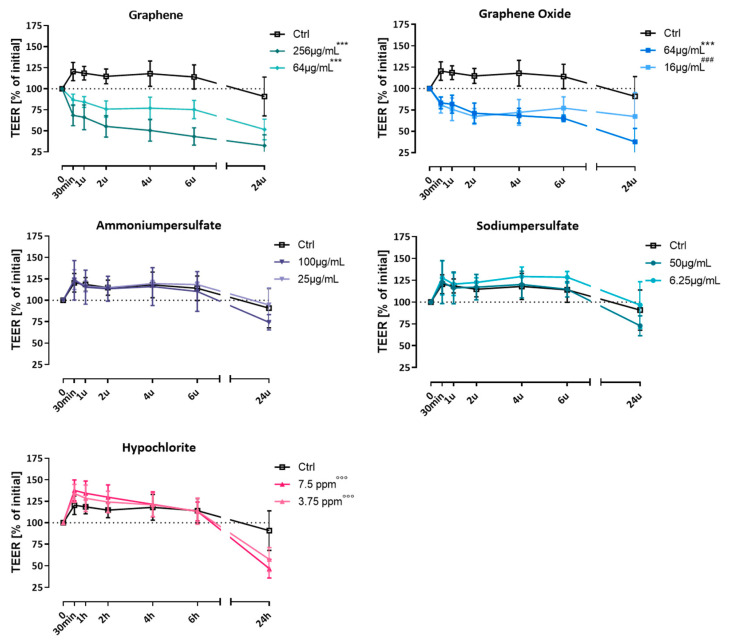
TEER measurements of cocultures exposed to G, GO, AP, SP and ClO^−^—the TEER values are normalized and represented as percentages of the baseline value. Significant different at * all time points, at ^#^ some time points and at ° 24 h after the start of exposure compared to the vehicle control. *p* < 0.001: 3 symbols. Mean ± SD, *n* = 3–7, each experiment was performed in duplicate.

TEER was recorded immediately before and at different time points (30 min, 1, 2, 4, 6 and 24 h) after the start of the exposure. Exposure to each tested concentration of G resulted in an immediate and persistent decrease in TEER compared to the vehicle-exposed control (Figure 3). Exposure to GO also induced a significant immediate decrease in TEER, which stayed significantly lower than the control for a concentration of 64 µg/mL (Figure 3). When the cocultures were exposed to AP or SP this did not result in changes in TEER compared to the control coculture (Figure 3). Both concentrations of active chlorine in ClO^−^ initially did not alter the TEER, yet 24 h after the start of the exposure a significant decrease in TEER compared to the control was found (Figure 3).

### 3.2. Flux of Fluorescent-Labelled Dextran

**Figure 4:**
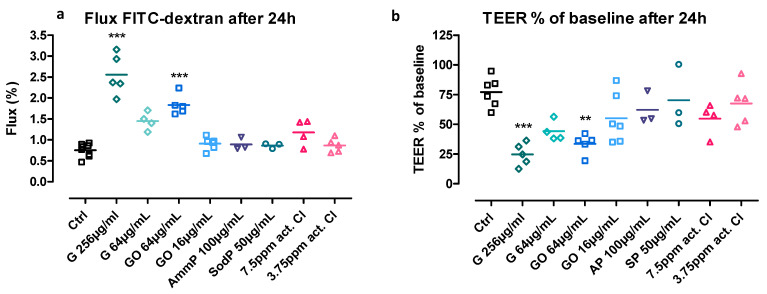
FITC-dextran transport (**a**) and TEER (**b**) of cocultures exposed to G, GO, SP, AP and ClO^−^—** *p* < 0.01, *** *p* < 0.001 compared to the vehicle control. Mean ± SD, *n* = 3–8; each experiment was performed in duplicate.

To investigate whether environmental irritants can alter the paracellular transport of macromolecules, we determined the flux of an apically added fluorescent-labelled 4 kDa-dextran (FD4) across the cell layers. The cocultures were exposed identically as described above for 20 h, after which the TEER was recorded and the flux was measured (after a 4 h incubation period to allow passage). Permeability for FD4 was significantly larger than in the control condition after exposure to 256 µg/mL of G and 64 µg/mL of GO (Figure 4a). Other tested concentrations of G and GO, and both concentrations ClO^−^, AP and SP did not significantly alter the FD4-flux after 24 h of exposure. The significant changes in FD4-flux were accompanied by significant decreases in the TEER (Figure 4b).

### 3.3. Effect of GNPs on the Airway Epithelium-Staining of Tight Junction (TJ) Proteins

**Figure 5:**
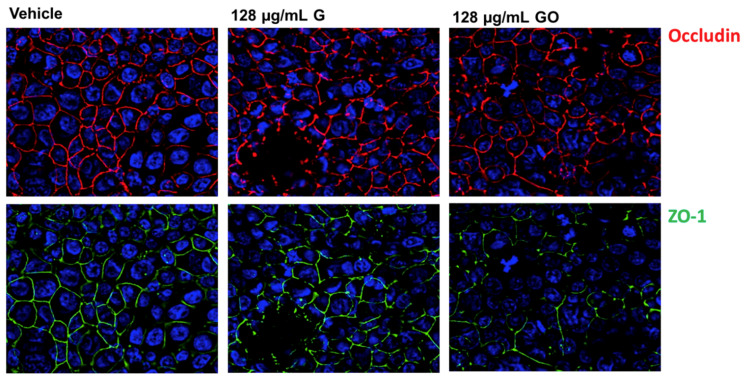
Immunofluorescent staining of TJ in cocultures exposed to G and GO suspended using 0.2% FCS. Representative images from cocultures apically exposed to vehicle, 128 µg/mL graphene and 128 µg/mL graphene oxide suspended using 0.2% FCS. Immunofluorescent staining for Occludin (red) and ZO-1 (green), cell nuclei were counterstained with DAPI (blue).

To investigate whether G or GO exposure disturbed the intercellular junctional complexes, the cocultures were immunostained for two tight junction markers, zonula occludens 1 (ZO-1) and occludin. Twenty-four hours after the start of the exposure, the vehicle-treated control coculture showed an intact TJ network with nicely connected ZO-1 and occludin staining (Figure 5). After exposure to 128 µg/mL G and GO in the FCS suspension, the TJ proteins were still present, but showed a more disorganized pattern with interruptions in the ZO-1 and occludin staining (Figure 5).

### 3.4. Phosphoproteomics of Membrane-Associated Proteins

Since the phosphorylation of intercellular junctional proteins might influence their functioning, and thus the barrier integrity, we investigated the phosphorylation level of the membrane-associated proteins after 24 h of exposure to 64 µg/mL G and GO suspended with FCS. Of the large number of identified phosphopeptides, those that had a relevant alteration in phosphorylation level (i.e., a NP-exposed versus/Ctrl phosphopepetide abundance ratio ≤ 0.5 or ≥ 1.5) were taken into consideration. For the cells exposed to G, we identified 242 unique phosphoproteins that had a relevant change in phosphorylation level compared to the control. In cells exposed to GO, 246 phosphoproteins were correspondingly identified. Per type of exposure, the identified differently phosphorylated proteins were functionally analyzed to determine their contribution in pathways associated with barrier integrity. As demonstrated in Table 1, the top five, based on *p*-value, out of the 15 significantly enriched KEGG pathways (*p*-value < 0.05) for G included ‘Spliceosome’, ‘Adherens junction’, ‘RNA transport’, ‘Ribosome biogenesis in eukaryotes’ and ‘Tight junction’. For GO, the top 5 out of 17 significantly enriched KEGG pathways included ‘Spliceosome’, ‘Tight junction’, ‘Adherens junction’, ‘RNA transport’ and ‘Cell adhesion molecules’, as listed in Table 2.

**Figure 6:**
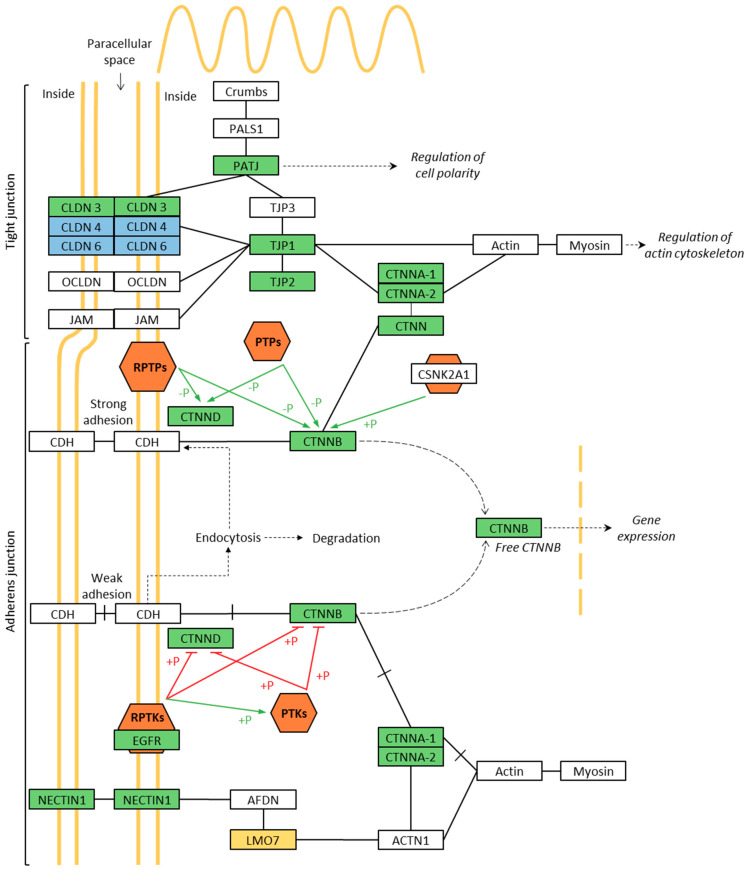
Overview of differentially phosphorylated proteins in TJ and AJ pathways—the proteins that were differently phosphorylated due to exposure to G, GO or both GNPs are depicted, respectively, in yellow, blue or green boxes. Proteins that were unaffected are depicted in white boxes. The orange hexagons represent protein kinases or phosphatases. ‘+P’ and ‘−P’ indicate, respectively, phosphorylation and de-phosphorylation; if this activates the function of the targeted protein a green arrow is drawn, if this inhibits the function of the targeted protein a red arrow is drawn. RPTP, receptor protein tyrosine phosphatase; PTP, protein tyrosine phosphatase; RTPK, receptor protein tyrosine kinase; PTK, protein tyrosine kinase. The figure is a simplified and combined version of the TJ and AJ pathway from the KEGG database.

Figure 6 shows a simplified and combined version of the TJ and AJ pathways provided from the KEGG database. The 15 differently phosphorylated proteins in the TJ and AJ pathways due to G, GO or both GNP exposures are depicted in colored boxes. TJ are essential to establish cell polarity and create a selective permeable barrier. The proteins Crumbs, PALS1 and PATJ constitute together the cell polarity complex in epithelial cells. TJs establish cell–cell contacts via transmembrane proteins of the claudin, occludin or junctional adhesion molecules (JAM) family and a cytoplasmic ‘plaque’ consisting of different proteins that form a complex and connect to the actin cytoskeleton. AJs are responsible for cellular adhesion in different types of tissues, thereby maintaining tissue architecture and limiting cell movement and proliferation. Cadherin is an essential cell adhesion molecule in AJ. The cytoplasmic tail of cadherin binds β-catenin, which in turn binds α-catenin, which connects to the actin cytoskeleton. Catenin-δ stabilizes the cadherin/β-catenin complex at the cell membrane. Phosphorylation of β-catenin and δ-catenin by receptor protein tyrosine kinases (RPTKs) and cytoplasmic protein tyrosine kinases (PTKs, e.g., Fer, Fyn, Yes and Src), negatively affects the integrity of the cadherin/catenin complex, leading to its dissociation. The integrity of this complex is positively regulated by dephosphorylation by protein tyrosine phosphatases and, by phosphorylation by casein kinase II. Changes in β-catenin phosphorylation affects cell–cell adhesion and influences the cellular location of β-catenin, which, if not associated with cadherin, can degrade or signal as free β-catenin to initiate gene expression. Nectins also function as cell adhesion molecules and transduce signals that reorganize the actin cytoskeleton, regulate AJ formation and strengthen cell–cell adhesion.

## 4. Discussion

As evaluated by TEER and FD4-flux, the nanoparticles G and GO immediately had a negative impact on the epithelial barrier, whereas ClO^−^ only had a negative impact after 24 h of exposure. Exposure to AP and SP did not alter the barrier properties. Next, evaluation of the TJ network using immunofluorescent staining of ZO-1 and occludin, indicated that cocultures exposed to G or GO had a slightly less connected TJ network. In a large set of membrane-associated proteins, we found using phosphoproteomics that the proteins in the AJ and TJ pathways were significantly hypo- or hyperphosphorylated due to GNP exposure, which therefore provides an explanation for the observed negative effect on the airway epithelial barrier.

An intact epithelial barrier is crucial, since disruption will enhance the permeability, which will lead to increased penetration of potentially harmful agents towards the host. Therefore, in the first part of this study, we evaluated the effect of GNPs and compared our observations to other environmental agents on the airway epithelial barrier. It was found that the GNPs had an immediate and persistent negative effect on the airway epithelial barrier. In comparison, the oxidizing chemicals sodium and ammonium persulfate did not have a negative effect on the barrier using the applied concentrations. Exposure to hypochlorite, on the other hand, impaired the barrier after 24 h of exposure. Transepithelial electrical resistance (TEER) measures the passage of ions and small molecules across the cell layers, whereas the flux of fluorescent-labeled dextran determines the paracellular transport of macromolecules [34]. In our experiments, these two parameters corresponded well and showed significant changes in FD4-flux and TEER for the highest tested concentration of G and GO. According to the WST-1 and LDH assay to determine cytotoxicity due to environmental exposure in 16HBE, these concentrations were non-cytotoxic.

In a second part, we further wanted to investigate the interaction of the GNPs with the epithelium. To investigate whether G and GO could destroy the intercellular junctions, we stained for the TJ proteins occludin and ZO-1. These are important TJ proteins that surround epithelial cells in a belt-like manner. After exposure to G and GO, the organization of the TJ belts was slightly altered, and still clearly present. The fact that the deposited G and GO particles appear black on the images makes it difficult to distinguish ‘disorganization of the TJ network’ from the black particles. Attempts to quantify our observations on the images were therefore unsuitable, making this only an indicative finding. In addition, not only the expression of the intercellular junctional proteins, but also their post-translational modifications (PTMs) determine the proper functioning of these proteins.

In a way to screen for alterations in a large set of proteins of the intercellular junctions, we performed a phosphoproteome analysis. Phosphorylation is a dynamic and important PTM that has been shown to regulate folding, binding, location and stability of intercellular junction proteins [35]. Using a phosphoproteome analysis on the membrane-associated proteins, we could evaluate a large set of proteins, providing us substantial information on the alterations due to NP exposure. Selecting the proteins that were significantly hypo- or hyperphosphorylated indicated that graphene oxide altered a slightly larger number of proteins than graphene.

The analysis revealed specific enrichment for spliceosome components and that of ribosome biogenesis for both G and GO, as these pathways are tightly coupled. Changes in similar pathways have been observed for crude oil on human airway epithelial cells, where RNA-seq analyses revealed enrichment in ribosomal biosynthesis (hsa03008) and spliceosome (hsa03040) [36]. Interestingly ribosome biogenesis has also been linked to oncogenic signaling, including that of c-Myc [37,38]. Transcriptome analysis of peripheral blood leukocytes in children with therapy-resistant asthma (SA) and controlled persistent asthma has revealed enrichment of pathways associated with ribosomal proteins, RNA transport and the spliceosome [39]. Also, in the context of development of severe asthma, KEGG enrichment of the spliceosome (mRNAs; SF3A1, SNRPE, SF3B4) has been observed [40]. Specific proteins in the KEGG pathway included ACIN1, serine/arginine-rich splicing factors, and Ubiquitin specific peptidase, the differential expression of which have been associated with altered cell proliferation and poor cancer prognosis, among others [41,42,43]. Another specific protein, SRSF6 (serine and arginine rich splicing factor 6), observed in our enrichment analysis, has also been shown to be dysregulated in ASM cells from asthmatic horses [44].

Functional analysis of these selected proteins also revealed that for both G and GO, the KEGG pathways AJ and TJ were found in the top five of significantly enriched pathways, which was in line with our hypothesis that GNP exposure might affect AJ and TJ protein functions. The enrichment in AJ and TJ pathways is specifically relevant, as such enrichment has also been observed in the Balb/c mice model (eosinophilic, mixed, and neutrophilic) of asthma from whole-genome transcriptome profiling of the lung, with enrichment in airway TJ [45]. For some specific proteins in these pathways, phosphorylation of specific residues is crucial in their function to maintain cell adhesion via interaction with different proteins (see Figure 6). Phosphorylation of tyrosine residues in occludin, E-cadherin, ZO-1 and β-catenin have repeatedly been associated with an increased barrier permeability, because of a reduced interaction between the proteins [35,46,47]. In our dataset of differentially phosphorylated AJ and TJ proteins, tyrosine phosphorylation was only found at Tyr865 in catenin δ-1, which is as expected because low abundant phosphotyrosine phosphorylation is more efficiently captured by immunoaffinity enrichment than by the more generalistic IMAC enrichment that was used for this study [2,48]. This is a known phosphorylated residue, which, however, has not been described to cause barrier alterations. Not only tyrosine phosphorylation is of importance for interactions between proteins of the AJ or TJ pathway. Phosphorylation of serine at position 641 in α-catenin has been described to disturb the binding between α-catenin and β-catenin, resulting in increased transcriptional activity of β-catenin, as well as reduced association of the cadherin/β-catenin complex with the actin skeleton [49]. In our experiments, G exposure increased the phosphorylation at Ser641 in catenin α-1, which might result in ‘looser’ AJs that contribute to the decreased barrier function. Similarly, we found increased serine phosphorylation at residues 552 and 191 in β-catenin, which has been shown to cause its dissociation from the cell–cell contacts and stabilization of β-catenin in the nucleus, respectively, and thus could be involved in the observed impaired barrier function [50,51].

While such interpretation may be limited due to the present study design, based on evidence presented in the literature, it can be said that enrichment of pathways such as ‘Ribosome biogenesis’, ‘RNA transport’, ‘Tight junction’, ‘Adherens junction’, ‘RNA transport’ and ‘Cell adhesion molecules’ may also be indicative of a possible Epithelial-Mesenchymal transition (EMT) that may contribute to airway remodeling in asthma [52,53,54,55].

## 5. Conclusions

In conclusion, in an in vitro model of the airway epithelial barrier, the nanoparticles G and GO had a negative impact on the barrier properties. To explain the negative impact of GNPs on the epithelial barrier, we evaluated whether the TJ proteins ZO-1 and occludin were affected. A less organized TJ network was observed after exposure to G and GO, but since the deposited GNP appeared black on the images, this observation was difficult to verify. Using phosphoproteomics, we revealed that the phosphorylation status of proteins of the AJ and TJ pathway changed due to GNP exposure, which could explain the observed negative impact of the GNPs on the epithelial barrier.

## Figures and Tables

**Table 1:** Functional classification of the differentially phosphorylated proteins by graphene exposure (fold change ≥ 1.5 or ≤ 0.5). Fifteen of the enriched pathways had a *p*-value < 0.05. The table lists the top 5 out of these enriched pathways.

No.	Full Gene Name	Gene ID	*p*-Value	Enrichment	FDR
	Spliceosome		6.92 × 10^−13^	10.28	8.15 × 10^−10^
1	Apoptotic chromatin condensation inducer 1	ACIN1			
2	Calcium homeostasis endoplasmic reticulum protein	CHERP			
3	DEAH-box helicase 16	DHX16			
4	Heterogeneous nuclear ribonucleoprotein C (C1/C2)	HNRNPC			
5	Heterogeneous nuclear ribonucleoprotein K	HNRNPK			
6	Pre-mRNA processing factor 38A	PRPF38A			
7	RNA binding motif protein 17	RBM17			
8	RNA binding motif protein 8A	RBM8A			
9	X-linked RNA binding motif protein X-linked	RBMX			
10	Squamous cell carcinoma antigen recognized by T-cells 1	SART1			
11	Small nuclear ribonucleoprotein U5 subunit 200	SNRNP200			
12	Serine and arginine rich splicing factor 4	SRSF4			
13	Serine and arginine rich splicing factor 6	SRSF6			
14	Serine and arginine rich splicing factor 9	SRSF9			
15	THO complex 2	THOC2			
16	Transformer 2 alpha homolog	TRA2A			
17	Transformer 2 beta homolog	TRA2B			
18	Ubiquitin specific peptidase 39	USP39			
	**Adherens Junction**		**3.40 × 10^−5^**	**8.56**	**0.04**
1	Catenin alpha 1	CTNNA1			
2	Catenin alpha 2	CTNNA2			
3	Catenin beta 1	CTNNB1			
4	Catenin delta 1	CTNND1			
5	Epidermal growth factor receptor	EGFR			
6	Lim domain 7	LMO7			
7	Nectin cell adhesion molecule 1	NECTIN1			
8	Tight junction protein 1	TJP1			
	**RNA Transport**		**3.72 × 10^−4^**	**4.41**	**0.44**
1	Apoptotic chromatin condensation inducer 1	ACIN1			
2	FMR1 autosomal homolog 2	FXR2			
3	Nucleoporin 88	NUP88			
4	Nucleoporin 93	NUP93			
5	Nucleoporin 98	NUP98			
6	Pinin desmosome associated protein	PNN			
7	RNA binding motif protein 8A	RBM8A			
8	Serine and arginine repetitive matrix 1	SRRM1			
9	THO complex 2	THOC2			
10	THO complex 5	THOC5			
	**Ribosome Biogenesis in Eukaryotes**		**8.95 × 10^−4^**	**6.11**	**1.05**
1	Dyskerin pseudouridine synthase 1	DKC1			
2	NOP56 ribonucleoprotein	NOP56			
3	NOP58 ribonucleoprotein	NOP58			
4	Treacle ribosome biogenesis factor 1(TCOF1)	TCOF1			
5	UTP 14 small subunit processome component	UTP14A			
6	UTP 18 small subunit processome component 18	UTP18			
7	5′-3′ exoribonuclease 2	XRN2			
	**Tight Junction**		**1.96 × 10^−3^**	**4.43**	**2.29**
1	Claudin 3	CLDN3			
2	Catenin alpha 1	CTNNA1			
3	Catenin alpha 2	CTNNA2			
4	Catenin beta 1	CTNNB1			
5	Cortactin	CTTN			
6	Crumbs cell polarity complex component	PATJ			
7	Tight junction protein 1	TJP1			
8	Tight junction protein 2	TJP2			

**Table 2:** Functional classification of differentially phosphorylated proteins by graphene oxide exposure (fold change ≥ 1.5 or ≤ 0.5). Seventeen of the enriched pathways had a *p*-value < 0.05. The table lists the top 5 out of these enriched pathways.

No.	Full Gene Name	Gene ID	*p*-Value	Enrichment	FDR
	Spliceosome		1.75 × 10^−12^	9.74	2.08 × 10^−9^
1	Apoptotic chromatin condensation inducer 1	ACIN1			
2	Calcium homeostasis endoplasmic reticulum protein	CHERP			
3	DEAH-box helicase 16	DHX16			
4	Heterogeneous nuclear ribonucleoprotein C (C1/C2)	HNRNPC			
5	Heterogeneous nuclear ribonucleoprotein K	HNRNPK			
6	Pre-mRNA processing factor 38A	PRPF38A			
7	RNA binding motif protein 8A	RBM8A			
8	X-linked RNA binding motif protein X-linked	RBMX			
9	Squamous cell carcinoma antigen recognized by T-cells 1	SART1			
10	Small nuclear ribonucleoprotein U5 subunit 200	SNRNP200			
11	Serine and arginine rich splicing factor 10	SRSF10			
12	Serine and arginine rich splicing factor 4	SRSF4			
13	Serine and arginine rich splicing factor 6	SRSF6			
14	Serine and arginine rich splicing factor 9	SRSF9			
15	THO complex 2	THOC2			
16	Transformer 2 alpha homolog	TRA2A			
17	Transformer 2 beta homolog	TRA2B			
18	Ubiquitin specific peptidase 39	USP39			
	**Tight Junction**		**1.01 × 10^−4^**	**5.25**	**0.12**
1	Claudin 3	CLDN3			
2	Claudin 4	CLDN4			
3	Claudin 6	CLDN6			
4	Catenin alpha 1	CTNNA1			
5	Catenin alpha 2	CTNNA2			
6	Catenin beta 1	CTNNB1			
7	Cortactin	CTTN			
8	Crumbs cell polarity complex component	PATJ			
9	Tight junction protein 1	TJP1			
10	Tight junction protein 2	TJP2			
	**Adherens Junction**		**4.02 × 10^−4^**	**7.10**	**4.75 × 10^−1^**
1	Catenin alpha 1	CTNNA1			
2	Catenin alpha 2	CTNNA2			
3	Catenin beta 1	CTNNB1			
4	Catenin delta 1	CTNND1			
5	Epidermal growth factor receptor	EGFR			
6	Nectin cell adhesion molecule 1	NECTIN1			
7	Tight junction protein 1	TJP1			
	**RNA Transport**		**5.57 × 10^−4^**	**4.18**	**0.66**
1	Apoptotic chromatin condensation inducer 1	ACIN1			
2	Eukaryotic translation initiation factor 5B	EIF5B			
3	FMR1 autosomal homolog 2	FXR2			
4	Nucleoporin 93	NUP93			
5	Nucleoporin 98	NUP98			
6	Pinin desmosome associated protein	PNN			
7	RNA binding motif protein 8A	RBM8A			
8	Serine and arginine repetitive matrix 1	SRRM1			
9	THO complex 2	THOC2			
10	THO complex 5	THOC5			
	**Cell Adhesion Molecules (CAM)**		**7.04 × 10^−4^**	**4.56**	**0.83**
1	Claudin 3	CLDN3			
2	Claudin 4	CLDN4			
3	Claudin 6	CLDN6			
4	Major histocompatibility complex class I A	HLA-A			
5	Major histocompatibility complex class I B	HLA-B			
6	Major histocompatibility complex class I C	HLA-C			
7	L1 cell adhesion molecule	L1CAM			
8	Myelin protein zero like 1	MPZL1			
9	Nectin cell adhesion molecule 1	NECTIN1			
